# Dr. H. Whitaker

**Published:** 1912-06-08

**Authors:** 


### Obituary.

Dr. H. Whitaker, M.D., of Belfast, a well-known member of the medical profession in Ireland, has died at the age of seventy-five. Dr. Whitaker, who graduated at Queen's University in 1859, had been superintendent medical officer for the City of Belfast for nearly twenty years. He studied at Queen's College, Belfast, and became a lecturer on sanitary science, and qualified M.R.C.S. in 1857.

